# Women’s experiences and views on early breastfeeding during the COVID-19 pandemic in Norway: quantitative and qualitative findings from the IMAgiNE EURO study

**DOI:** 10.1186/s13006-023-00553-5

**Published:** 2023-03-10

**Authors:** Eline Skirnisdottir Vik, Sigrun Kongslien, Ingvild Hersoug Nedberg, Ilaria Mariani, Emanuelle Pessa Valente, Benedetta Covi, Marzia Lazzerini

**Affiliations:** 1 Department of Health and Caring Sciences, Western Norway University of Applied Sciences, Bergen, Norway; 2 Department of Health and Care Sciences, UiT The Arctic University of Norway, Tromsø, Norway; 3 Institute for Maternal and Child Health, IRCCS “Burlo Garofolo”, WHO Collaborating Centre for Maternal and Child Health, Trieste, Italy

**Keywords:** Early breastfeeding, COVID-19, Questionnaire, Survey, Respectful maternity care, Quality of maternal and newborn care, Norway, IMAgiNE EURO

## Abstract

**Background:**

Little is known about women’s experience of care and views on early breastfeeding during the COVID-19 pandemic in Norway.

**Methods:**

Women (*n* = 2922) who gave birth in a facility in Norway between March 2020 and June 2021 were invited to answer an online questionnaire based on World Health Organization (WHO) Standard-based quality measures, exploring their experiences of care and views on early breastfeeding during the COVID-19 pandemic. To examine associations between year of birth (2020, 2021) and early breastfeeding-related factors, we estimated odds ratios (ORs) with 95% confidence intervals (CIs) using multiple logistic regression. Qualitative data were analysed using Systematic Text Condensation.

**Results:**

Compared to the first year of the pandemic (2020), women who gave birth in 2021 reported higher odds of experiencing adequate breastfeeding support (adjOR 1.79; 95% CI 1.35, 2.38), immediate attention from healthcare providers when needed (adjOR 1.89; 95% CI 1.49, 2.39), clear communication from healthcare providers (adjOR 1.76; 95% CI 1.39, 2.22), being allowed companion of choice (adjOR 1.47; 95% CI 1.21, 1.79), adequate visiting hours for partner (adjOR 1.35; 95% CI 1.09, 1.68), adequate number of healthcare providers (adjOR 1.24; 95% CI 1.02, 1.52), and adequate professionalism of the healthcare providers (adjOR 1.65; 95% CI 1.32, 2.08). Compared to 2020, in 2021 we found no difference in skin-to-skin contact, early breastfeeding, exclusive breastfeeding at discharge, adequate number of women per room, or women’s satisfaction. In their comments, women described understaffed postnatal wards, early discharge and highlighted the importance of breastfeeding support, and concerns about long-term consequences such as postpartum depression.

**Conclusions:**

In the second year of the pandemic, WHO Standard-based quality measures related to breastfeeding improved for women giving birth in Norway compared to the first year of the pandemic. Women’s general satisfaction with care during COVID-19 did however not improve significantly from 2020 to 2021. Compared to pre-pandemic data, our findings suggest an initial decrease in exclusive breastfeeding at discharge during the COVID-19 pandemic in Norway with little difference comparing 2020 versus 2021. Our findings should alert researchers, policy makers and clinicians in postnatal care services to improve future practices.

**Supplementary Information:**

The online version contains supplementary material available at 10.1186/s13006-023-00553-5.

## Background

The COVID-19 pandemic has affected maternal and perinatal healthcare worldwide [1, 2]. New and continuously shifting government regulations had a large impact on the organization of hospital care, including restrictions that may have affected breastfeeding [3, 4]. Both the World Health Organization (WHO) and the International Confederation of Midwives (ICM) called the attention of the international community to women’s and newborn’s rights during the COVID-19 pandemic [5, 6]. However, a reduction in exclusive breastfeeding initiation during the first wave of the pandemic was observed [3, 7]. Further, European hospitals have differed in their interpretation of guidance around breastfeeding policies during the pandemic, with some advising formula feeding although no national or international guidelines recommended discontinuation of breastfeeding [8].

Breastfed children are less likely to die of infections [9], less likely to become overweight [10] and are less prone to diabetes and allergy later in life [11], while their intelligence quotients can be higher [12, 13]. Women who breastfeed have a reduced risk of a range of physical and emotional health problems, such as ovarian and breast cancers, postpartum depression and maternal excess of stress [14]. The multiple benefits of breastfeeding likely outweigh possible risk factors related to SARS-CoV-2 infection and should therefore be encouraged [15, 16]. In addition, while SARS-CoV-2 transmission from mother to baby via breastmilk seems to be unlikely, milk produced by infected mothers has been found to be a source of anti-SARS-CoV-2 IgA and IgG valuable for the baby’s health [16].

This study is part of the IMAgiNE EURO project, a multi-country survey conducted in 18 countries in the WHO European Region to collect views of women on the quality of maternal and newborn healthcare during the COVID-19 pandemic [17]. Similar to what occurred in other European countries, in order to prevent the spread of COVID-19, since 12 March 2020 the Norwegian government introduced strict social distancing regulations with major impacts on society [18]. During the COVID-19 pandemic in Norway, an increased use of formula, a reduction in exclusive breastfeeding and reduction in the average length of breastfeeding were documented [4]. However, little is known about women’s experience of care and views on early breastfeeding during the COVID-19 pandemic in Norway. More evidence on this topic may be of interest for researchers, policy makers and clinicians and support further improvements in the quality of maternal and newborn care.

The aim of this study was to investigate quality of care at facility level, and women’s experiences and views on early breastfeeding practices during different phases of the COVID-19 pandemic in Norway.

## Methods

### Study design

Details of the multi-country IMAgiNE EURO project are reported elsewhere [17, 19]. In the current mixed-method cross-sectional study, both quantitative and qualitative data were analysed to investigate women’ experiences and views on early breastfeeding during the pandemic. To strengthen the reporting of quantitative data, a STROBE (Strengthening the reporting of observational studies in epidemiology) checklist was used (see Additional File 1). Likewise, we used the COREQ Checklist (COnsolidated criteria for REporting Qualitative research Checklist) in reporting our qualitative data (see Additional File 2).

### Setting

In Norway, approximately 53,000 babies are born each year [20]. Maternity care is part of the public healthcare system and is built on the principle of free and equal access for all regardless of factors such as ethnicity or social background [21, 22]. Prior to the COVID-19 pandemic, Norway reported one of the highest rates of exclusive breastfeeding at 6 months of age in Europe [23]. Breastfeeding is promoted in a national action plan launched in 2017 for a healthier diet [24] and national surveys published in 2020 showed that 78% of babies in Norway were breastfed at 6 months [25] and 48% at 12 months [26]. By National law, parents in Norway are entitled to 49 weeks paid parental leave [27]. Additionally, a nursing mother returning to work is entitled to 30 minutes time off, which may be taken twice daily or as a reduction in working hours by up to 1 hour per day, to promote breastfeeding [28].

During the COVID-19 pandemic, in Norway the risk of maternal hospitalization due to COVID-19 disease in pregnancy was low [29]. However, maternity wards changed their practices, companion of choice often encountered restrictions in participation of care [30], and women were on average discharged from hospital earlier than in preceding years [20]. Further, during the pandemic women who gave birth in Norway have described feelings of loneliness and isolation in relation to antenatal care, when arriving at the hospital for labor, in cases of induction of labor, and at the postnatal ward [30]. This could result in a lack of maternal support which is crucial for the establishment of exclusive breastfeeding, especially during the hospital stay in the early postpartum period [3]. Women remained isolated from their social network after discharge due to strict social distancing regulations [18]. While several restrictions remained in healthcare services in Norway in 2021, the first year of the pandemic (2020) was characterized by more uncertainties both for new families and healthcare workers.

### Participants

**Fig. 1:**
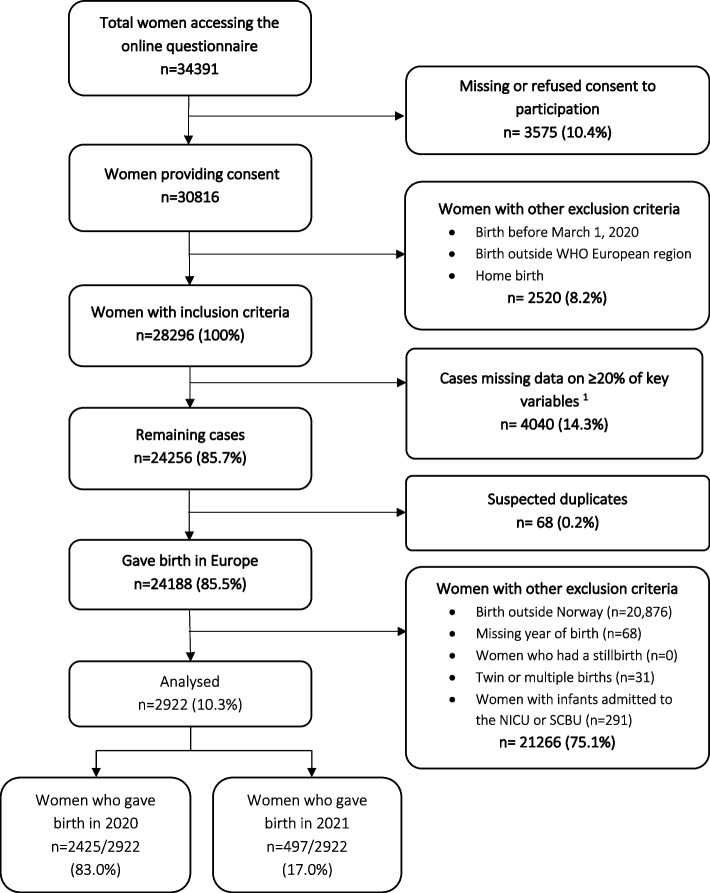
Flowchart of the derivation of the study sample. Women who gave birth in Europe during the COVID-19 pandemic (March 2020 to July 2021) ^1^Percentage of missing data for each woman was calculated over mandatory questions (*n* = 45)

Data were collected with a voluntary anonymous online survey, open to women ≥18 years-of-age who gave birth at a facility in the WHO European Region between 1 March 2020 and 30 June 2021 (*n* = 34,391). Data were cleaned as previously reported according to standard operating procedures [17]. Briefly, suspected duplicates and cases missing 20% or more answers on the 40 key quality measures and five key socio-demographic variables (i.e., date of birth, age, education, parity, whether the women gave birth in the same country where she was born) were excluded. The current study includes responses given by women who gave birth in Norway in the same period (*n* = 2922). For the purposes of this study, twin or multiple births (*n* = 31), and infants admitted to the Neonatal Intensive Care Unit (NICU) or Special Care Baby Unit (SCBU) (*n* = 291) were excluded from the analysis. Further exclusion of cases can be seen in Fig. 1.

### Data collection

Data were collected with an online questionnaire (see Additional File 3). The questionnaire was developed and validated by an international team of experts [17]. The questionnaire was built on WHO standards of improving quality of maternal and newborn care (QMNC) (*n* = 30) [31] and also included questions relevant due to the COVID-19 pandemic (*n* = 10). The wording on education levels was agreed among partners during a Delphi exercise including 26 experts from 11 countries of the WHO European Region [17, 19]. The last question in the questionnaire was an open-ended question with no word limit to collect input and suggestions from mothers. The open-ended question was phrased as follows: *Do you have any suggestions to improve quality of care provided at the facility where you gave birth or to improve this questionnaire?*

The online survey was made available in 22 languages and actively promoted though social media (i.e., Facebook groups, such as pregnancy due date groups, migrant groups in Norway and a group for breastfeeding mothers) in Norway by researcher ESV. Project partners in the WHO European Region promoted the survey through social media, organizational websites, local networks and Non-Governmental Organizations by project partners [17]. Women participated in the study in their preferred language regardless of which country they gave birth in.

Data were collected through an online survey using REDCap 8.5.21 (© 2021 Vanderbilt University).

### Data analysis

Both quantitative and qualitative data were analysed. In the current study, the analysis of quantitative data included 13 out of the original 40 quality measures in the full survey. All variables (*n* = 3) explicitly describing breastfeeding and infant feeding were based on WHO’s standards for improving QMNC in health facilities [31]. In addition, qualitative data were extracted from the final open-ended question collecting women’s suggestions on how to improve quality of care. Both quantitative and qualitative variables are described in the following text.

### Analysis of quantitative data

We used descriptive statistics to summarize quantitative data, reported as frequencies and percentages. Differences of sample characteristics by year of birth (2020, 2021) were tested with a Chi-square test or a Fisher exact test. To examine associations between year of birth and early breastfeeding-related factors (*n* = 13), we estimated crude and adjusted odds ratios (adjORs) with 95% confidence intervals (CIs) using logistic regression. The main exposure was year of birth (2020, 2021). Outcome variables were dichotomized variables related to early breastfeeding and included the following: opportunity to have skin-to-skin within the first hour after birth (yes, no); early breastfeeding within the first hour after giving birth when applicable (yes, no); adequate breastfeeding support (yes, no); exclusive breastfeeding at discharge from hospital (yes, no); immediate attention by healthcare providers when needed (yes, no); clear communication from healthcare providers (yes, no); allowed companion of choice (yes, no); adequate number of women per room (yes, no); adequate visiting hours for companion of choice (yes, no); adequate number of healthcare providers (yes, no); adequate professionalism of the healthcare providers (yes, no); reduction in QMNC due to COVID-19 (yes, no); and reduction in their general satisfaction due to COVID-19 pandemic (yes, no). When the option “partially” was available, such answers were categorized as “no”. Other variables included in the model were: women born in Norway (yes, no, missing); answered the survey in other language than Norwegian (yes, no); age range (18–24, 25–30, 31–35, 36–39, ≥ 40); educational level (None, Elementary school, Junior High school, High School, University degree, Postgraduate degree / Master / Doctorate or higher); parity (1, > 1); birth mode (vaginal spontaneous, instrumental vaginal birth, Cesarean section).

A two-tailed *P*-value < 0.05 was considered statistically significant. Statistical analyses were performed using Stata version 14 (Stata Corporation, College Station, TX, USA) and R version 4.1.1 (R Foundation for Statistical Computing, Vienna, Austria).

### Analysis of qualitative data

Systematic Text Condensation (STC) was used to analyse data related to breastfeeding from the open-ended question. Comments including variations on the words breastfeeding and milk / formula, suckle / latch, and / or breast were included in the qualitative analysis. STC was conducted using the following four step method: 1) We became familiar with the data and identified preliminary themes (i.e., *partner*, *information*, *understaffed ward*, *consequences of poor breastfeeding support*, *positive descriptions of breastfeeding support*, and *poor competence*); 2) Meaning units describing women’s experiences and views on early experiences with breastfeeding during the COVID-19 pandemic in Norway were sorted into code groups (i.e., *postnatal wards*, *partner’s role*, and *consequences of poor breastfeeding support*); 3) Code groups were sorted into subgroups, and meaning units in each subgroup were summarized and condensed; and 4) The condensates from Step 3 formed the basis for our final analytic text [32]. In line with the method and guidelines for reporting qualitative research, we used direct quotations to elucidate the findings [32]. Initially, qualitative data were analysed by one researcher (ESV) using NVivo (NVivo Release 1.6.1 (4830), 1999-2022 QSR International). In Step 3, preliminary results were discussed with two more researcher, SK and IHN, who both had access to the raw data. ESV, SK and IHN read and agreed on the final analytic text.

## Results

The study sample included answers from 2922 births, 2425 (83.0%) in 2020 and 497 (17.0%) in 2021 were included (Fig. 1). Characteristics of the study sample are shown in Table 1. Both in 2020 and 2021, few women had an immigrant background (*n* = 204) or answered the survey in a different language than Norwegian (*n* = 19). In both 2020 and 2021, most women were 25 to 35 years old; nearly four out of five had either a university or postgraduate degree; the number of instrumental births was similar (12.4% vs. 11.5%). When compared to 2021, the numbers of first-time mothers were statistically significant higher in 2020 (58.4% vs. 53.1%, *P* = 0.030); Cesarean sections were slightly but not statistically significant more frequent (14.3% vs. 11.3%); and the number of women born outside Norway and vaginal spontaneous births were slightly but not statistically significantly lower (7.2% vs. 5.8 and 73.3% vs. 77.3%, respectively).

**Table 1:** Characteristics of the study sample

Year of birth	2020 ***N*** = 2425	2021 ***N*** = 497	***P***-value
**Women born in Norway**			
Yes	2249 (92.7)	468 (94.2)	0.258
No	175 (7.2)	29 (5.8)	0.271
Missing	1 (0.0)	0 (0.0)	> 0.99
**Answered the survey in a language other than Norwegian**			
Yes	17 (0.7)	2 (0.4)	0.654
No	2408 (99.3)	495 (99.6)	0.654
**Age range**			
18–24	147 (6.1)	32 (6.4)	0.750
25–30	1059 (43.7)	237 (47.7)	0.101
31–35	910 (37.5)	184 (37.0)	0.833
36–39	249 (10.3)	37 (7.4)	0.054
≥ 40	60 (2.5)	7 (1.4)	0.148
**Educational level**^**a**^			
None	1 (0.0)	0 (0.0)	> 0.99
Elementary school	0 (0.0)	0 (0.0)	–
Junior High school	37 (1.5)	7 (1.4)	0.845
High School	498 (20.5)	101 (20.3)	0.914
University degree	1244 (51.3)	259 (52.1)	0.741
Postgraduate degree / Master / Doctorate or higher	645 (26.6)	130 (26.2)	0.839
**Parity**			
1	1416 (58.4)	264 (53.1)	0.030
> 1	1009 (41.6)	233 (46.9)	0.030
**Birth mode**			
Vaginal spontaneous	1778 (73.3)	384 (77.3)	0.068
Instrumental vaginal birth	301 (12.4)	57 (11.5)	0.559
Cesarean section	346 (14.3)	56 (11.3)	0.077

^a^Wording on education levels was agreed among partners during the Delphi exercise

Associations between year of birth (2020, 2021) and factors related to early breastfeeding (*n* = 13 factors) are shown in Table 2. When compared to the first year of the pandemic (2020), women who gave birth in 2021 reported higher odds of adequate breastfeeding support (adjOR 1.79; 95% CI 1.35, 2.38); immediate attention when needed (adjOR 1.89; 95% CI 1.49, 2.39); clear communication from healthcare providers (adjOR 1.76; 95% CI 1.39, 2.22); allowed companion of choice (adjOR 1.47; 95% CI 1.21, 1.79); adequate visiting hours for partner and / or relatives (adjOR 1.35; 95% CI 1.09, 1.68); adequate number of healthcare providers (adjOR 1.24; 95% CI 1.02, 1.52); and adequate professionalism of the healthcare providers (adjOR 1.65; 95% CI 1.32, 2.08). When compared to the first year of the pandemic (2020), women who gave birth in 2021 reported lower odds of reduction in QMNC due to COVID-19 pandemic (adjOR 0.73; 95% CI 0.60, 0.89). We found no difference by year of birth; in having skin-to-skin contact with the baby in the first hour after giving birth; early breastfeeding; exclusive breastfeeding at discharge; adequate number of women per room; or reduction in their general satisfaction due to COVID-19.

**Table 2:** Results of the logistic regression analysis^a^

Factors related to early breastfeeding	n cases (yes)	n women (total)	Crude OR (95% CI)	Adjusted OR^**b**^ (95% CI)
**Opportunity to have skin-to-skin contact in the first hour after giving birth** ***(when applicable,*** **i.e.** ***, in absence of maternal or neonatal health problems)***				
2020	2148	2270	1.00	1.00
2021	460	480	1.31 (0.81, 2.12)	1.07 (0.59, 1.92)
**Early breastfeeding** ***(when applicable, i.e., in absence of maternal or neonatal health problems)***				
2020	1968	2233	1.00	1.00
2021	430	469	1.48 (1.04, 2.11)	1.32 (0.88, 1.96)
**Adequate breastfeeding support**				
2020	1902	2425	1.00	1.00
2021	433	497	1.86 (1.40, 2.46)	1.79 (1.35, 2.38)
**Exclusive breastfeeding at discharge**				
2020	1868	2425	1.00	1.00
2021	402	497	1.26 (0.99, 1.61)	1.14 (0.89, 1.46)
**Immediate attention when needed**				
2020	1636	2425	1.00	1.00
2021	397	497	1.91 (1.51, 2.42)	1.89 (1.49, 2.39)
**Clear communication from healthcare providers**				
2020	1657	2425	1.00	1.00
2021	395	497	1.79 (1.42, 2.27)	1.76 (1.39, 2.22)
**Allowed companion of choice**				
2020	962	2425	1.00	1.00
2021	246	497	1.49 (1.23, 1.71)	1.47 (1.21, 1.79)
**Adequate number of women per room**				
2020	1812	2425	1.00	1.00
2021	356	497	0.85 (0.69, 1.06)	0.84 (0.67, 1.04)
**Adequate visiting hours for partner and / or relatives**				
2020	552	2425	1.00	1.00
2021	141	497	1.34 (1.08, 1.67)	1.35 (1.09, 1.68)
**Adequate number of healthcare providers**				
2020	830	2425	1.00	1.00
2021	198	497	1.27 (1.04, 1.55)	1.24 (1.02, 1.52)
**Adequate professionalism of the healthcare providers**				
2020	1594	2425	1.00	1.00
2021	380	497	1.69 (1.35, 2.12)	1.65 (1.32, 2.08)
**Reduction in the QMNC due to COVID-19**				
2020	1631	2425	1.00	1.00
2021	294	497	0.71 (0.58, 0.86)	0.73 (0.60, 0.89)
**Reduction in their general satisfaction due to COVID-19** ***(among women who reported a reduction in the QMNC due to COVID-19)***				
2020	1442	1631	1.00	1.00
2021	264	294	1.15 (0.77, 1.73)	1.13 (0.75, 1.70)

^a^OR are calculated with 2020 year of birth as reference category

^b^Analyses were adjusted for the following variables: born in Norway (yes, no, missing), responded to a non-Norwegian survey (yes, no), age range (18–24, 25–30, 31–35, 36–39, ≥ 40), educational level (None, Elementary school, Junior high school, High school, University degree, Postgraduate degree / Master / Doctorate or higher), parity (1, > 1), birth mode (Vaginal spontaneous, Instrumental vaginal birth, Cesarean section). Wording on education levels agreed among partners during the Delphi exercise

Abbreviations: *COVID-19* Corona virus disease of 2019, *QMNC* Quality of maternal and newborn care

### Findings on qualitative data

Of the 2922 women who responded, 1021 (34.9%) provided a free-text comment. Of the 1021 free-text comments, 88 comments (8.6%) were directly related to breastfeeding. There were eight non-Norwegian language comments (i.e., four comments in English, three in Swedish and one in German; none of which were related to breastfeeding). The following themes were identified during analysis of the open-ended question: 1) Understaffed postnatal wards, 2) Early discharge and a lack of professional support, 3) The importance of breastfeeding support from companion of choice, and 4) Long term consequences.

### Understaffed postnatal wards

Women in the study described understaffed postnatal wards. Positive characterizations of healthcare providers included words such as being nice and skilled, often followed by descriptions of how the wards were understaffed and unavailable to the women. One woman who pointed out that understaffed postnatal wards may be a common problem, put it like this:

*“The breastfeeding guidance was extremely poor and divergent*. *.. A large part of the staff was nice, but it was very clear that they were understaffed. For all I know, understaffing [of postnatal wards] is a common problem.” (Norwegian woman No. 1746).*

### Early discharge and a lack of professional support

Among negative narratives, several mentioned that they would have liked to stay for longer at the postnatal ward, to learn how to breastfeed and feel safe before being discharged. Some related early discharge due to COVID-19 restrictions and explained how they had to be discharged early to lessen the risk of spreading the virus. Others disclosed voluntary early discharge due to COVID-19 restrictions or a lack of help at the hospital. Most birth narratives contained positive language, while descriptions of the immediate postpartum period tended to be more negative in nature. Women in the study highlighted a need for improved breastfeeding support and continuity of care. Most women described intentions to breastfeed as a means of providing optimal nutrition and to bond with the newborn. Some women described being advised to watch videos or use the internet for breastfeeding support, while others described healthcare providers as stressed or heavy-handed when asking for breastfeeding support. Women reported feeling like a burden, being forgotten, or feeling like bad mothers. Some described how they felt pushed to breastfeed by healthcare providers and not being offered help if they for different reasons preferred to abstain from breastfeeding. Others described how healthcare providers fed the baby with formula without information or consent. Some women questioned the healthcare providers competence in breastfeeding and the quality of handovers between hospital shifts. One woman who initially planned to breastfeed, who ended up not breastfeeding, described it like this:

*“The staff often said, ‘I’ll get someone to help you’, but it never happened. It happened several times that I had to wait more than half an hour to get help. With a screaming newborn that I was unable to breastfeed, it felt absolutely horrific. The staff would come, but then had to run again halfway through a sentence.” (Norwegian woman No. 552).*

### The importance of breastfeeding support from companion of choice

While some women described how partners were allowed to stay at the postnatal ward, other women described how their companion of choice was not allowed to visit her and baby at the postnatal ward due to COVID-19 restrictions. Some women discharged themselves early to be reunited with their partner. Having a partner present during the postnatal period was identified as a supportive factor for breastfeeding. This was reported to have positive impacts on the emotional well-being of the mother, as well as providing practical assistance and reducing the workload on healthcare providers. One woman who preferred her partner to stay at the postnatal ward put it like this:

*“It was a great burden that my partner was not allowed to be there. Having children and breastfeeding is a family project, and not something I had volunteered to do alone.” (Norwegian woman No. 3135).*

Others were pleased with the calm atmosphere that followed the strict visitor restrictions, including restrictions related to the partner. A calm atmosphere was described as positive for breastfeeding.

### Long term consequences

Women expressed how family centered care was lacking, with possible negative consequences for breastfeeding, bonding, and becoming a family. Further, fearing postpartum depression was mentioned by several, and some stated that their experiences made them fear having more children in the future. One woman who believes she suffers from a postpartum depression put it in the following way:

*“..*. *I experienced the first days of my daughter’s life as very traumatic. Due to COVID-19 restrictions, we also did not receive sufficient help and guidance (including breastfeeding support). Due to the lack of help, I have struggled with what I believe is a postpartum depression.” (Norwegian woman No. 506).*

## Discussion

When compared to women who gave birth in Norway during the first year of the COVID-19 pandemic, women who gave birth the following year were more likely to experience adequate breastfeeding support; immediate attention when needed; clear communication from healthcare providers; being allowed a companion of choice; adequate visiting hours for partner and / or relatives; adequate number of healthcare providers; and adequate professionalism of the healthcare providers. Compared to 2020, in 2021, the women also experienced lower odds of reduction in QMNC due to COVID-19 pandemic. When comparing factors related to breastfeeding in 2020 vs. 2021, we found no difference in the opportunity to have skin-to-skin contact with the baby in the first hour after giving birth; early breastfeeding; exclusive breastfeeding at discharge; adequate number of women per room; or reduction in their general satisfaction due to COVID-19. In comments related to breastfeeding (2020 and 2021), women described understaffed postnatal wards; early discharge and highlighted the importance of breastfeeding support from healthcare providers and companion of choice; and concerns about long-term consequences such as postpartum depression.

Many factors contribute to successful breastfeeding in the early postpartum period, and we found a range of breastfeeding-related factors were improved for women giving birth in Norway in 2021, compared to the first year of the COVID-19 pandemic (2020). One UK study from the COVID-19 pandemic reported that face-to-face breastfeeding support was reduced during the pandemic and some women struggled to get breastfeeding support, while others found strict regulations positive because of increased time at home, less pressure and fewer visitors [33]. In the current study, answers to the open-ended question revealed some women were pleased with the calm atmosphere that followed the strict visitor restrictions. Our findings are in line with the UK study and suggest that the COVID-19 pandemic affected women’s breastfeeding experiences differently.

When comparing data from 2020 with data from 2021, we found little difference in early skin-to-skin contact, early breastfeeding within the first hour after giving birth or exclusive breastfeeding at discharge. Women’s satisfaction with the number of women per room stayed constant over the study period and women’s general satisfaction with care due to COVID-19 did not improve significantly from 2020 to 2021. One Italian study including 204 mothers and babies in the early stage of the COVID-19 pandemic (9 March to 8 May 2020) found a decrease in exclusively breastfeeding in the studied population [3]. Consistent with our findings, one study including 821 women who gave birth in Norway in the spring of 2020 found great reliance on breast-milk substitutes, which may imply that fewer women in Norway were exclusively breastfeeding during the initial phase of the COVID-19 pandemic [4]. Findings of a quantitative study including 3642 women giving birth in Norway during the pandemic adds support that one in three women experienced being discharged early due to COVID-19 related factors [34]. In our data, one in four women (23.2%) who underwent labor in Norway in the study period reported not exclusively breastfeeding at discharge [17]. In the current study, when comparing data from 2020 with data from 2021, we found no difference in early breastfeeding or exclusive breastfeeding at discharge. In 2020, a nation-wide Norwegian report showed that 97% of babies born in Norway in 2018 were breastfed before postpartum discharge [25]. To our knowledge, updated national data on exclusive breastfeeding at discharge has not been published. However, early discharge may be one explanation for why fewer women exclusively breastfeed their babies early in the pandemic [4]. The lack of improvement in exclusive breastfeeding during the study period should alert policy makers in postnatal care services to implement specific quality improvement actions. To better understand the reasons for a lack of improvement in exclusive breastfeeding at discharge and no change in women’s general satisfaction with care due to COVID-19 from 2020 to 2021, future studies with other designs are needed.

In comments related to breastfeeding, the open-ended question in our survey gave information on understaffed postnatal wards, the importance of breastfeeding support from healthcare professionals and companion of choice, and a concern for long term consequences, such as postpartum depression, due to insufficient breastfeeding support during the pandemic. Our findings related to understaffed postnatal wards and the importance of partner are supported by a qualitative study exploring women’s experiences with giving birth in Norway during the pandemic [30]. Further, studies from Norway and the UK support the concern for the occurrence of postpartum depression, as they found an increase in maternal depression and anxiety postpartum, during the COVID-19 pandemic [34, 35]. A Norwegian national report on parental experience of QMNC published before the COVID-19 pandemic (2018) found that new parents in Norway were well satisfied with the care given on labor wards, however, postnatal care scored lower than other areas [36]. Findings in the Norwegian report suggest that our results related to QMNC in Norway during the first year of the Covid-19 pandemic cannot be attributed to the pandemic alone and must therefore be interpreted with caution. The qualitative findings in the current study support the concerns arising from the quantitative data, such as those related to women experiencing inadequate breastfeeding support, lack of attention when needed, not being allowed companion of choice, or a low number of healthcare providers.

### Strengths and limitations

It may be seen as a limitation to the study that changes in local and national COVID-19 regulations over time were not accounted for. Because we only included data from women who gave birth during the pandemic, comparison with pre-pandemic data must be made with caution. It may be seen as a strength that the study includes both quantitative and qualitative data (i.e., triangulation or mixed-methods), an approach which provides a more comprehensive picture of the results than either method could do alone [37]. The qualitative data is not suitable for quantification, and comparison between the free-text responses given in 2020 vs. 2021 is therefore not included. The study used standard procedures and indicators, and allowed for future rounds of data collection, and comparison over time and settings. Open-ended questions can provide crucial information that closed-ended questions cannot deliver [38, 39]. Women themselves chose whether to answer the open-ended question or not, thus these questions were not subject to systematic measurements [38, 39]. Therefore, we did not analyse the open-ended questions for 2020 and 2021 separately. Due to self-administration, open-ended questions may cause selection bias in those responding [39]; women who were satisfied with breastfeeding support may be less likely to provide comments related to breastfeeding. The results from the open-ended questions should therefore be interpreted with caution. We acknowledge that the online survey lacked important information on the sample, such as more information on maternal and newborn clinical characteristics which may be relevant for the interpretation of the results [17]. Caution is necessary when comparing the current study’s 7.0% response rate for migrant women with national data indicating that 28.9% of women who gave birth in Norway in 2020 and 2021 were born outside the country [20]. Women who experienced vaginal birth, planned or emergency Cesarean sections were all included in analysis, however, experiences related to early breastfeeding may differ between these groups due to several factors. Causality cannot be drawn from this cross-sectional study [40].

### Recommendation for research and for policies

Our study provides critical information for researchers, policy makers and clinicians on the need for continuous surveillance of national breastfeeding rates and for improving postnatal care services and breastfeeding support in Norway and similar settings. This study highlights the importance of promoting continuity of care and evidence-based interventions, such as inclusion of companion of choice in postnatal wards. To improve women’s general satisfaction with postpartum care, adequate staffing for breastfeeding support must be made available to all new mothers.

## Conclusions

In the second year of the COVID-19 pandemic, several but not all breastfeeding-related factors improved for women giving birth in Norway compared to the first year of the pandemic. Women’s general satisfaction with care during COVID-19 and rates of exclusive breastfeeding did however not improve significantly from 2020 to 2021. The findings should alert researchers, policy makers and clinicians in postnatal care services to improve future practices.

## Supplementary Information

**Additional file 1.**

**Additional file 2.**

**Additional file 3.**

## Data Availability

The datasets analysed during the current study are available from the corresponding author on reasonable request.

## Abbreviations

CI: Confidence intervals
COREQ: COnsolidated criteria for REporting Qualitative research
COVID-19: Coronavirus disease of 2019
ICM: International Confederation of Midwives
Ig: Immunoglobulin
NICU: Neonatal intensive care unit
OR: Odds ratio
QMNC: Quality of maternal and newborn care
SCBU: Special care baby unit
STC: Systematic Text Condensation
STROBE: Strengthening the reporting of observational studies in epidemiology
WHO: World Health Organization
