# Supplementary material for: Women’s experiences and views on early breastfeeding during the COVID-19 pandemic in Norway: quantitative and qualitative findings from the IMAgiNE EURO study
Source: Int Breastfeed J. 2023 Mar 10;18:15. doi: 10.1186/s13006-023-00553-5 (PMC9998246; doi:10.1186/s13006-023-00553-5)
Supplement: Supplementary file 1 — Additional file 1. [file 13006_2023_553_MOESM1_ESM.pdf]

STROBE Statement—checklist of items that should be included in reports of observational studies

	Item No.	Recommendation	Page No.	Relevant text from manuscript
**Title and abstract**	1	(*a*) Indicate the study's design with a commonly used term in the title or the abstract	Title page	Women's experiences and views on early breastfeeding during the COVID-19 pandemic in Norway: quantitative and qualitative findings from the IMAgiNE EURO Study
		(*b*) Provide in the abstract an informative and balanced summary of what was done and what was found	Page 2-3	Lines: 20-47
**Introduction**				
Background/rationale	2	Explain the scientific background and rationale for the investigation being reported	Page 4-5	Lines: 52-79
Objectives	3	State specific objectives, including any prespecified hypotheses	Page 5	Lines: 80-81
**Methods**				
Study design	4	Present key elements of study design early in the paper	Page 5	Lines: 84-86
Setting	5	Describe the setting, locations, and relevant dates, including periods of recruitment, exposure, follow-up, and data collection	Page 5-7	Lines: 90-140
Participants	6	(*a*) *Cohort study*—Give the eligibility criteria, and the sources and methods of selection of participants. Describe methods of follow-up<br>*Case-control study*—Give the eligibility criteria, and the sources and methods of case ascertainment and control selection. Give the rationale for the choice of cases and controls<br>*Cross-sectional study*—Give the eligibility criteria, and the sources and methods of selection of participants	Page 6-9	Lines: 114-187
		(*b*) *Cohort study*—For matched studies, give matching criteria and number of exposed and unexposed		

		Case-control study—For matched studies, give matching criteria and the number of controls per case		
Variables	7	Clearly define all outcomes, exposures, predictors, potential confounders, and effect modifiers. Give diagnostic criteria, if applicable	Page 8	Lines: 148-168
Data sources/ measurement	8*	For each variable of interest, give sources of data and details of methods of assessment (measurement). Describe comparability of assessment methods if there is more than one group	Page 7-9	Lines: 175-187
Bias	9	Describe any efforts to address potential sources of bias	Page 6 and 16	Lines: 117-123, 342-364
Study size	10	Explain how the study size was arrived at	Page 6-7, Figure 1	Lines: 114-116, 119-123

Continued on next page

Quantitative variables	11	Explain how quantitative variables were handled in the analyses. If applicable, describe which groupings were chosen and why	Page 7-8	Lines: 142-171
Statistical methods	12	(*a*) Describe all statistical methods, including those used to control for confounding	Page 8	Lines: 164-168
		(*b*) Describe any methods used to examine subgroups and interactions	n/a	
		(*c*) Explain how missing data were addressed	Page 6	Lines: 117-119
		(*d*) *Cohort study*—If applicable, explain how loss to follow-up was addressed<br>*Case-control study*—If applicable, explain how matching of cases and controls was addressed<br>*Cross-sectional study*—If applicable, describe analytical methods taking account of sampling strategy	n/a	
		(*e*) Describe any sensitivity analyses	n/a	
**Results**				
Participants	13*	(a) Report numbers of individuals at each stage of study—eg numbers potentially eligible, examined for eligibility, confirmed eligible, included in the study, completing follow-up, and analysed	Page 6-7, Figure 1	Lines: 114-123
		(b) Give reasons for non-participation at each stage	n/a	
		(c) Consider use of a flow diagram	Figure 1	
Descriptive data	14*	(a) Give characteristics of study participants (eg demographic, clinical, social) and information on exposures and potential confounders	Table 1	Lines: 189-198
		(b) Indicate number of participants with missing data for each variable of interest	Table 1	
		(c) *Cohort study*—Summarise follow-up time (eg, average and total amount)	n/a	
Outcome data	15*	*Cohort study*—Report numbers of outcome events or summary measures over time	n/a	
		Case-control study—Report numbers in each exposure category, or summary measures of exposure	n/a	
		Cross-sectional study—Report numbers of outcome events or summary measures	Table 2	
Main results	16	(*a*) Give unadjusted estimates and, if applicable, confounder-adjusted estimates and their precision (eg, 95% confidence interval). Make clear which confounders were adjusted for and why they were included	Table 2	
		(*b*) Report category boundaries when continuous variables were categorized	Table 1	Lines: 165-166
		(*c*) If relevant, consider translating estimates of relative risk into absolute risk for a meaningful time period	n/a	

Continued on next page

Other analyses	17	Report other analyses done—eg analyses of subgroups and interactions, and sensitivity analyses	n/a	
**Discussion**				
Key results	18	Summarise key results with reference to study objectives	Page 16	Lines 278-290
Limitations	19	Discuss limitations of the study, taking into account sources of potential bias or imprecision. Discuss both direction and magnitude of any potential bias	Page 18-19	Lines 342-364
Interpretation	20	Give a cautious overall interpretation of results considering objectives, limitations, multiplicity of analyses, results from similar studies, and other relevant evidence	Page 16-18	Lines 277-340
Generalisability	21	Discuss the generalisability (external validity) of the study results	Page 18-19	Lines 343-344, 351-364
**Other information**				
Funding	22	Give the source of funding and the role of the funders for the present study and, if applicable, for the original study on which the present article is based	Page 20-21	Lines 395-397

*Give information separately for cases and controls in case-control studies and, if applicable, for exposed and unexposed groups in cohort and cross-sectional studies.

Note: An Explanation and Elaboration article discusses each checklist item and gives methodological background and published examples of transparent reporting. The STROBE checklist is best used in conjunction with this article (freely available on the Web sites of PLoS Medicine at http://www.plosmedicine.org/, Annals of Internal Medicine at http://www.annals.org/, and Epidemiology at http://www.epidem.com/). Information on the STROBE Initiative is available at www.strobe-statement.org.
