# Supplementary material for: Women’s experiences and views on early breastfeeding during the COVID-19 pandemic in Norway: quantitative and qualitative findings from the IMAgiNE EURO study
Source: Int Breastfeed J. 2023 Mar 10;18:15. doi: 10.1186/s13006-023-00553-5 (PMC9998246; doi:10.1186/s13006-023-00553-5)
Supplement: Supplementary file 3 — Additional file 3. [file 13006_2023_553_MOESM3_ESM.pdf]

IMAGINE EURO
Improving MAternal Newborn carE

International Project

IMAGINE EURO

(Improving MAternal Newborn carE in the European Region)

Online survey on the preparedness, quality and resilience of the maternal and newborn health service among countries of the WHO European Region during the COVID-19 pandemic

The survey is based on ***WHO Standards for improving quality of maternal and newborn hospital care***

IMAgiNE EURO is coordinated by the WHO Collaborating Centre for Maternal and Child Health of Burlo Garofolo Hospital, Trieste (Italy)

Did you give birth during COVID-19 pandemic?

Help us improve the quality of maternity services in your country: take part at the international research study and tell us your experience of birth.

Your participation is voluntary: It will take approximately **15 minutes**

The survey is b*ased on WHO Standards for improving maternal and newborn hospital care.*

Unfortunately, the questionnaire is not adjusted to bereaved families.
If you have experienced the heavy loss of a child before its birth, or during infancy, it is important that you receive support.
You can access resources here: https://www.sands.org.uk/

The survey is anonymously: we're committed to your privacy.
To understand more about the processing of collected data and what we do with the data, refer to our privacy policy available at the link below. ________

Providing consent at the following question I declare that I am 18 years old or older and I voluntarily decide to participate at the research study.

CONSENT TO PARTICIPATION:

I have read and understand the above consent form, and, by selecting "Yes" below, I declare I am 18 years old or older and I indicate my willingness to voluntarily take part in the research study.
Yes □ No □

If NO:
By participating, your experience of giving birth may contribute for improving the quality of maternity services in your country.
It will take approximately 15 minutes.

S1. Did you give birth in a hospital after 1st March 2020 up to now? Yes □ No □
If NO:
Thank you for your interest in our research.
To participate is necessary to have given birth after 1st March 2020. If you know someone who did it, please share the link with her.
We wish you all the best.

S2. Where did you give birth? (Country & Region)
Please choose the country here / Please choose the Region here

1, Albania
2, Andorra
3, Armenia
4, Austria
5, Azerbaijan
6, Belarus
7, Belgium
8, Bosnia Herzegovina (→ **S2b.**)
9, Bulgaria
10, Croatia (→ **S2c.**)
11, Cyprus
12, Czech Republic
13, Denmark
14, Estonia
15, Finland
16, France (→ **S2f.**)
17, Georgia
18, Germany (→ **S2g.**)
19, Greece
20, Hungary
21, Iceland
22, Ireland
23, Israel
24, Italy (→ **S2i.**)
25, Kazakhstan
26, Kyrgyzstan
27, Latvia
28, Lithuania
29, Luxembourg
30, Macedonia
31, Malta
32, Monaco
33, Montenegro
34, Netherlands
35, Norway (→ **S2n.**)
36, Poland
37, Portugal (→ **S2p.**)
38, Republic of Moldova
39, Romania (→ **S2r.**)
40, Russian Federation
41, San Marino
42, Serbia
43, Slovakia
44, Slovenia
45, Spain (→ **S2sp.**)
46, Sweden
47, Switzerland
48, Tajikistan

49, Turkey
50, Turkmenistan
51, Ukraine
52, UK (→ **S2uk.**)
53, Uzbekistan
54, I have not found my Country (→ **S3.**)

S2f. Please specify the department where you gave birth.
1, 01-Ain
2, 02-Aisne
3, 03-Allier
4, 04-Alpes-de-Haute-Provence
5, 05-Hautes-Alpes
6, 06-Alpes-Maritimes
7, 07- Ardèche
8, 08-Ardennes
9, 09-Ariège
10, 10-Aube
11, 11-Aude
12, 12-Aveyron
13, 13-Bouches-du-Rhône
14, 14-Calvados
15, 15-Cantal
16, 16-Charente
17, 17-Charente-Maritime
18, 18-Cher
19, 19-Corrèze
20, 2A-Corse-du-Sud
21, 2B-Haute-Corse
22, 21-Côte-d'Or
23, 22-Côtes d'Armor
24, 23-Creuse
25, 24-Dordogne
26, 25-Doubs
27, 26-Drôme
28, 27-Eure
29, 28-Eure-et-Loir
30, 29-Finistère
31, 30-Gard
32, 31-Haute-Garonne
33, 32-Gers
34, 33-Gironde
35, 34-Hérault
36, 35-Ille-et-Vilaine
37, 36-Indre
38, 37-Indre-et-Loire
39, 38-Isère
40, 39-Jura

41, 40-Landes
42, 41-Loir-et-Cher
43, 42-Loire
44, 43-Haute-Loire
45, 44-Loire-Atlantique
46, 45-Loiret
47, 46-Lot
48, 47-Lot-et-Garonne
49, 48-Lozère
50, 49-Maine-et-Loire
51, 50-Manche
52, 51-Marne
53, 52-Haute-Marne
54, 53-Mayenne
55, 54-Meurthe-et-Moselle
56, 55-Meuse
57, 56-Morbihan
58, 57-Moselle
59, 58-Nièvre
60, 59-Nord
61, 60-Oise
62, 61-Orne
63, 62-Pas-de-Calais
64, 63-Puy-de-Dôme
65, 64-Pyrénées-Atlantiques
66, 65-Hautes-Pyrénées
67, 66-Pyrénées-Orientales
68, 67-Bas-Rhin
69, 68-Haut-Rhin
70, 69-Rhône
71, 70-Haute-Saône
72, 71-Saône-et-Loire
73, 72-Sarthe
74, 73-Savoie
75, 74-Haute-Savoie
76, 75-Paris
77, 76-Seine-Maritime
78, 77-Seine-et-Marne
79, 78-Yvelines
80, 79-Deux-Sèvres
81, 80-Somme
82, 81-Tarn
83, 82-Tarn-et-Garonne
84, 83-Var
85, 84-Vaucluse
86, 85-Vendée
87, 86-Vienne
88, 87-Haute-Vienne

89, 88-Vosges
90, 89-Yonne
91, 90-Territoire de Belfort
92, 91-Essonne
93, 92-Hauts-de-Seine
94, 93-Seine-St-Denis
95, 94-Val-de-Marne
96, 95-Val-D'Oise
97, 971-Guadeloupe
98, 972-Martinique
99, 973-Guyane
100, 974-La Réunion
101, 975-Mayotte

S2p. Please specify the region where you gave birth.
1, Aveiro
2, Beja
3, Braga
4, Bragança
5, Castelo Branco
6, Coimbra
7, Corvo
8, Évora
9, Faial
10, Faro
11, Flores
12, Graciosa
13, Guarda
14, Leiria
15, Lisboa
16, Madeira
17, Pico
18, Portalegre
19, Porto
20, Porto Santo
21, Santa Maria
22, Santarém
23, São Jorge
24, São Miguel
25, Setúbal
26, Terceira
27, Viana do Castelo
28, Vila Real
29, Viseu

S2i. Please specify the region where you gave birth.
1, Abruzzo
2, Basilicata

3, Calabria
4, Campania
5, Emilia-Romagna
6, Friuli Venezia Giulia
7, Lazio
8, Liguria
9, Lombardia
10, Marche
11, Molise
12, Piemonte
13, Puglia
14, Sardegna
15, Sicilia
16, Toscana
17, Trentino-Alto Adige
18, Umbria
19, Valle d'Aosta
20, Veneto

S2sp. Please specify the CCAA (autonomous community) where you gave birth.
1, Andalusia
2, Aragon
3, Asturias
4, Balearic Islands
5, Canary
6, Cantabria
7, Castile-La Mancha
8, Castile and Leon
9, Catalonia
10, Autonomous Community of Madrid
11, Valencian Community
12, Extremadura
13, Galicia
14, La Rioja
15, Murcia
16, Navarre
17, Basque Country

S2uk. Please specify the Home Nation of the UK where you gave birth.
1, England
2, Northern Ireland
3, Scotland
4, Wales

S2r. Please specify the region where you gave birth.

1, Regiunea Nord Est (județe: Suceava, Bacău, Botoșani, Iași, Neamț, Suceava și Vaslui).
3, Regiunea Sud-Est (județe: Brăila, Buzău, Constanța, Galați, Tulcea și Vrancea).

5, regiunea Sud - Muntenia (județe: Argeș, Călărași, Dâmbovița, Giurgiu, Ialomița, Prahova și Teleorman).
4, Regiunea Sud-Vest Oltenia (județe: Dolj, Gorj, Mehedinți, Olt și Vâlcea).
5, Regiunea Vest (județe: Arad, Caraș-Severin, Hunedoara și Timiș).
6, Regiunea Nord-Vest (județe: Bihor, Bistrița-Năsăud, Cluj, Maramureș, Satu-Mare și Sălaj).
7, Regiunea Centru (județe: Alba, Brașov, Covasna, Harghita, Mureș și Sibiu).
8, Regiunea București – Ilfov

S2n. Please specify the region where you gave birth.
1, Helse Nord
2, Helse Midt
3, Helse Vest
4, Helse Sør-Øst

S2c. Please specify the region where you gave birth.
1, Grad Zagreb
2, Bjelovarsko-bilogorska županija
3, Brodsko-posavska županija
4, Dubrovačko-neretvanska županija
5, Istarska županija
6, Karlovačka županija
7, Koprivničko-križevačka županija
8, Krapinsko-zagorska županija
9, Ličko-senjska županija
10, Međimurska županija
11, Osječko-baranjska županija
12, Požeško-slavonska županija
13, Primorsko-goranska županija
14, Sisačko-moslavačka županija
15, Splitsko-dalmatinska županija
16, Šibensko-kninska županija
17, Varaždinska županija
18, Virovitičko-podravska županija
19, Vukovarsko-srijemska županija
20, Zadarska županija
21, Zagrebačka županija
22, Izvan Hrvatske
23, Nešto drugo

S2b. Please specify the region where you gave birth.
1, Tuzla Canton
2, Sarajevo Canton
3, Zenica-Doboj Canton
4, Una-Sana Canton
5, Central Bosnia Canton
6, Herzegovina-Neretva Canton
7, West Herzegovina Canton

8, Canton 10
9, Posavina Canton
10, Bosnian-Podrinje Canton
11, Banja Luka region
12, Prijedor Region
13, Doboj Region
14, Bijeljina Region
15, East Sarajevo Region
16, Trebinje region
17, Brcko District

S2g. Please specify the region where you gave birth.
1, Baden-Württemberg
2, Bavaria
3, Berlin
4, Brandenburg
5, Bremen
6, Hamburg
7, Hesse
8, Mecklenburg-Western Pomerania
9, Lower Saxony
10, North Rhine-Westphalia
11, Rhineland-Palatinate
12, Saarland
13, Saxony
14, Saxony-Anhalt
15, Schleswig-Holstein
16, Thuringia

S3. The research is exclusive to the WHO European region, which includes 53 countries, covering a vast geographical region from the Atlantic to the Pacific oceans.
If you can't find your country in the list, where are you from? Please specify

Section A - Hospital reception

A1. When you arrived at the hospital, did you feel you received attention within an appropriate amount of time (triage and waiting time)?

1) Yes
2) Just in part
3) No

Section B - Labour

B1. Did healthcare providers ask your permission before performing vaginal examinations on you?

1) Yes, always
2) Sometimes
3) No, never
4) I never had vaginal examinations

B2. Did you experience active labour (the phase of labour in which you felt strong, regular and painful contractions and the dilatation was around 4cm or greater)?

1) Yes (→**B3 and B4**)
2) No (→**Section C. Childbirth**)
3) I don't know (→**B3 and B4**)

B3. Once strong and regular contractions began, did healthcare providers allow you to move freely or were you asked to stay in bed?

1) Yes, I was allowed to move (e.g., get out of the bed, to walk, exercise, etc.)
2) In part, only sometimes they allowed me to move
3) No, they asked me to stay in the bed all the time, lying on my back or in the bed with variations to switch on my left/right thigh

B4. During labour and delivery, did you ask for and/or receive some kind of pain relief (including pharmacological options such as epidural or other medicines, and non-pharmacological options such as massages, warm bath)?

1) Yes, I requested them and they were given to me
2) Yes, I requested them but I was denied
3) No, I didn't ask for them but they were offered by the healthcare providers
4) No, I didn't ask for them and they weren't offered

Section C - Childbirth

C1. How was your baby born?

1) Spontaneous vaginal delivery (→**Vaginal Birth: C2 and C3**)
2) Instrumental vaginal delivery (by vacuum extraction or forceps) (→**Instrumental vaginal birth: C2 and C3**)
3) Emergency caesarean section during labour (→**Caesarean Section: C2 and C3**)
4) Emergency caesarean section before going into labour
5) Planned or elective caesarean section before going into labour

Vaginal Birth

C2. During the last phase of your labour, when the baby moves down through the vagina and is going to be born, did you feel free to choose your position, to find the most comfortable one for you?

1) Yes
2) Sometimes
3) No

C3. During your delivery, did healthcare providers make a cut/surgical incision in the perineum, known as an episiotomy?

1) Yes
2) No

Instrumental vaginal birth

C2. Did healthcare providers expressly request your consent to use one or both of these instruments (vacuum extraction and/or forceps)? *verbal consent is also fine
1) Yes
2) No

C3. And to facilitate childbirth of your baby, did healthcare providers exert pressure on your belly with hands/arms during labour to help you give birth? *(this manoeuvre is called “Kristeller”)
1) Yes
2) No

Caesarean section

C2. As soon as your child was born (while still in the operating theatre), did they give you information regarding his/her healthcare?
1) Yes
2) No

C3. After caesarean section, do you feel you received adequate pain relief?
1) Yes
2) No

Section D - After birth

D1. In the first hour after giving birth, did you have the opportunity to have skin-to-skin contact with your baby?
1) Yes
2) No
3) I didn't have the possibility to do it due to health-care problems (e.g., I had a post-partum haemorrhage, my baby was admitted to the NICU*, etc.)

*Neonatal Intensive Care Unit

D2. In the first hour after giving birth, did you try to breastfeed your baby?
1) Yes
2) No
3) I didn't have the possibility to do it due to health-care problems (e.g., I had a post-partum haemorrhage, my baby was admitted to the NICU*, etc.)

*Neonatal Intensive Care Unit

D3. Were you appropriately supported by healthcare providers to breastfeed your baby? (e.g., they helped you to initiate and establish breastfeeding and/or explained how to manage common initial breastfeeding difficulties, etc.)
1) Yes
2) No

D4. When they brought you to the ward, was your baby:
1) Always with me in the room, also during the night (rooming in)
2) Mostly with me in the room
3) Mostly at the nursery *Note: The nursery is the hospital department usually

dedicated to healthy newborns
4) Always at the nursery
5) Hospitalized in NICU (neonatal intensive care unit) or SCUBU (special care baby unit)

D5. While you were at the hospital after birth, were you allowed to stay with your baby as long as you wished?
1) Yes
2) No

D6. Did healthcare providers inform you exhaustively about possible clinical signs of alarm for you (e.g. Excessive vaginal bleeding, difficulty urinating, difficulty breathing)?
1) Yes
2) No

D7. Did healthcare providers inform you exhaustively about possible clinical signs of alarm for your child? (e.g., breathing difficulties, excessive sweat, tremors, neonatal jaundice)?
1) Yes
2) No

D8. How were you feeding your baby when you were discharged from the hospital?
1) Exclusively breastfeeding
2) Exclusively with infant formula by my personal choice
3) Exclusively with infant formula as prescribed by healthcare providers
4) A mix between breastfeeding and infant formula by my personal choice
5) A mix between breastfeeding and infant formula as prescribed by healthcare providers

Section E - Birth rights

E1. Did you feel that you received immediate attention from healthcare providers to be assisted when you needed?
1) Yes, always/ nearly always
2) Sometimes
3) No, never/almost never

E2. Did healthcare providers establish effective and clear communication with you? (e.g., did they speak clearly so that you understood/make you feel comfortable in the dialogue and in asking questions/checking that you understood)?
1) Yes, always/ nearly always
2) Sometimes
3) No, never/almost never

E3. Did healthcare providers fully involve you in the choices of care/treatment that you received? (e.g., did they give you clear explanations, respect your autonomy, ask for your preferences or opinions)
1) Yes, always/ nearly always
2) Sometimes
3) No, never/almost never

E4. Was your companion of choice allowed to stay with you for as long as you felt the need?

1) Yes, always/ nearly always
2) Sometimes
3) No, never/almost never

E5. Did you feel treated with dignity?

1) Yes, always/ nearly always
2) Sometimes
3) No, never/almost never

E6. During childbirth, did you feel emotionally supported (e.g., did healthcare providers show attention and empathy for your emotional state and offer support to make you feel better)?

1) Yes, always/ nearly always
2) Sometimes
3) No, never/almost never

E7. Did you feel that your privacy was protected by healthcare providers (e.g., at least curtains were used or you were covered up during examinations, did they discuss private information in a way that others could not hear)?

1) Yes, always/nearly always
2) Sometimes
3) No, never/almost never

E8. Did you suffer any forms of physical (e.g., have you been touched improperly and / or without asking your permission, have you been pushed, beaten, slapped, pinched, physically restrained or gagged), verbal (e.g., have you been shouted at, insulted or talked rudely), or emotional abuse (e.g., have you been neglected, mocked or forgotten by healthcare providers)?

1) Yes, always/ nearly always (→**E8a.**)
2) Sometimes (→**E8a.**)
3) No, never

E8a. Please, specify what kind of abuse did you suffer of: (choose all that apply)

1) Physical abuse (e.g., have you been touched improperly and / or without asking your permission, have you been pushed, beaten, slapped, pinched, physically restrained or gagged)
2) Verbal abuse (e.g., have you been shouted at, insulted or talked rudely)
3) Emotional abuse (e.g., have you been neglected, mocked or forgotten by healthcare providers)
4) Other (→E8b)

E8b. Please, specify

E9. During your hospitalization, did you make any kind of informal payment (e.g., money other than official costs, bribes or gifts) to access better quality services?

1) Yes, always/ nearly always
2) Sometimes
3) No, never/almost never

Section F - Overall satisfaction

Regarding your general satisfaction with the hospital where you gave birth, please rank the items below:

	Good/Excellent	Sufficient	Insufficient/Very bad
F1. Comfort and general equipment of the rooms for you and your baby			
F2. Number of women that you had to share the room with during recovery			
F3. Appropriate and regular cleaning of rooms			
F4. Appropriate number of bathrooms, adequately located, furnished and cleaned			
F5. Visiting hours for partner and/or relatives			
F6. Adequate number of healthcare providers considering the workload			
F7. Professionality of healthcare providers			

F8. Taking into account all the previous considerations that you made, how would you evaluate your experience in the hospital when you gave birth?

1) Very positive
2) Positive
3) Negative
4) Very negative

Section G – COVID-19 pandemic

G1. Due to the COVID-19 pandemic, did you have difficulties attending routine clinical checks during pregnancy (e.g., antenatal care visits, ultrasound checks, etc.)?

1) Yes, always/ nearly always
2) Sometimes
3) No, never/almost never

G2. Did you face any kind of barrier to access the facility where you were assisted during pregnancy? (e.g., logistic, financial, due to lock-down, lack of childcare for children at home etc.)

1) Yes, always/ nearly always
2) Sometimes
3) No, never/almost never

G3. Were there adequate infographics (i.e., posters, signs, images) present in the hospital in which you gave birth to indicate the path to follow or the rules to follow to reduce the risk of infection as much as possible?

1) Yes, they were present and adequate
2) Yes, they were present but in part adequate
3) No

G4. Do you think that the divisions between wards and common spaces were adequately reorganized to reduce the risk of COVID-19 infection as much as possible?

1) Yes, effectively

2) Yes, but in part adequate
3) No

G5. Do you think the hospital rooms were properly reorganized to reduce the risk of COVID-19 infection as much as possible? (e.g., reducing the number of hospitalized mothers in each room)

1) Yes, adequately
2) Yes, but in part adequate
3) No

G6. Did the facility where you gave birth have at least one functioning and easily accessible hand-washing station (near or inside your room) adequately supplied with water and soap or disinfectant alcohol solution?

1) Yes, adequately
2) Yes, but in part adequate
3) No

G7. Were healthcare providers always equipped with adequate personal protective equipment (masks, gloves) at the time of the visit and did they use them correctly?

1) Yes, always/ nearly always
2) Sometimes
3) No, never/almost never

G8. Were healthcare providers always present in sufficient numbers to guarantee adequate assistance despite the COVID-19 pandemic?

1) Yes, always/ nearly always
2) Sometimes
3) No, never/almost never

G9. Do you think that healthcare providers were able to use adequate communication methods to reduce as much as possible the stress related to new procedures required by the COVID-19 pandemic?

1) Yes, always/ nearly always
2) Sometimes
3) No, never/almost never

G10. In you experience, did you feel that you had any limitations in the quality of hospital care received due to COVID-19 pandemic?

1) Yes, always/ nearly always (→**G11.**)
2) Sometimes (→**G11.**)
3) No, never/almost never

G11. How did these limitations affect your satisfaction?

1) Improved it a lot
2) Improved it a little
3) Made it a little worse
4) Made it much worse
5) Did not affect my satisfaction

Section H-Socio demographic data

H1. When did you give birth?

Select month and year of birth (start selection from March 2020)

H2. Is this your first-born child?

1) Yes
2) No

H3. Did you give birth in the same country where you were born?

1) Yes
2) No

H4. What is your age? Please select a range.

1) 18-24
2) 25-30
3) 31-35
4) 36-39
5) >=40

H5. What is the highest level of education you have attained?

1) None (→**H5a.**)
2) Elementary school
3) Junior high school
4) High school
5) University degree
6) Post-graduate degree / Master / Doctorate or higher

H5a. Who helped you to answer this questionnaire?

1) My partner
2) A family member or friend
3) A healthcare provider
4) Other

H6. Have you experienced the following situations during your pregnancy, birth or postpartum (please, for postpartum consider only the period after birth when you were hospitalized)?
Choose all that apply.

1) I was tested for COVID-19 and the result was positive
2) I was tested for COVID-19 and the result was negative
3) I was tested for COVID-19 but I do not know the result yet or the result was inconclusive
4) Although I had respiratory symptoms, I was not tested for COVID-19 but I was clinically managed as a positive case
5) My baby was admitted to NICU (Neonatal Intensive Care Unit)
6) I was admitted to ICU (Intensive Care Unit)
7) I had a stillbirth
8) I had twins (or other multiple birth)
9) I was sent from one hospital to another during labour due to lack of beds or complications

10) Other, specify (→**H6a.**)
11) No, I did not experience any of those situations.

H6a. Please, specify.

H7. In what type of hospital did you give birth?
1) Public hospital
2) Private hospital/clinic

H7a. Who made the payment? Choose all that apply.
1) By public insurance/State or other non-private insurance (e.g., insurance covered by the company where you work)
2) By my private insurance
3) By me (or my family) without insurance

H8. Which kinds of healthcare providers directly assisted your birth? (choose all that apply)
1) Midwife
2) Nurse
3) A student (i.e., before graduation) (→**H8a.**)
4) Obstetrics registrar / medical resident (under post-graduation training)
5) Obstetrics and gynaecology doctor
6) I don't know (healthcare providers did not introduce themselves)
7) Other

H8a. What kind of student?
1) Midwife student
2) Nurse Student
3) Medical student

H9. How did you hear about this study (choose all that apply)?
1) Social media platform you engage (Facebook/Instagram)
2) Internet search (e.g., Google)
3) Received email from friend/family
4) Word of mouth
6) Press (e.g., radio, print media, TV)
5) Other

H10. Do you have any suggestions to improve quality of care provided at the facility where you gave birth or to improve this questionnaire?

Now you have finished,
We thank you so much for sharing with us your experience and for giving us your precious time.
Have a good day!
